# Splenogonadal Fusion: A Rare Mimicker of Malignancy

**DOI:** 10.1155/2022/2791305

**Published:** 2022-04-15

**Authors:** Natalie C. Hartman, Levent Trabzonlu, Güliz A. Barkan, Marcus L. Quek

**Affiliations:** ^1^Department of Urology, Loyola University Medical Center, Maywood, IL, USA; ^2^Department of Pathology, Loyola University Medical Center, Maywood, IL, USA

## Abstract

Splenogonadal fusion is a rare congenital anomaly in which ectopic splenic tissue is found in the testis and can present as a testicular mass mimicking a testicular malignancy. We present a 27-year-old male who presented with a palpable left testis mass suspicious for malignancy and ultimately found to have discontinuous splenogonadal fusion after radical orchiectomy.

## 1. Introduction

Splenogonadal fusion (SGF) is a congenital anomaly in which ectopic splenic tissue is located within or adjacent to a gonad. Splenic rests in the testicle are benign but can mimic a testicular malignancy and necessitate orchiectomy for diagnosis.

## 2. Case Presentation

**Figure 1:**
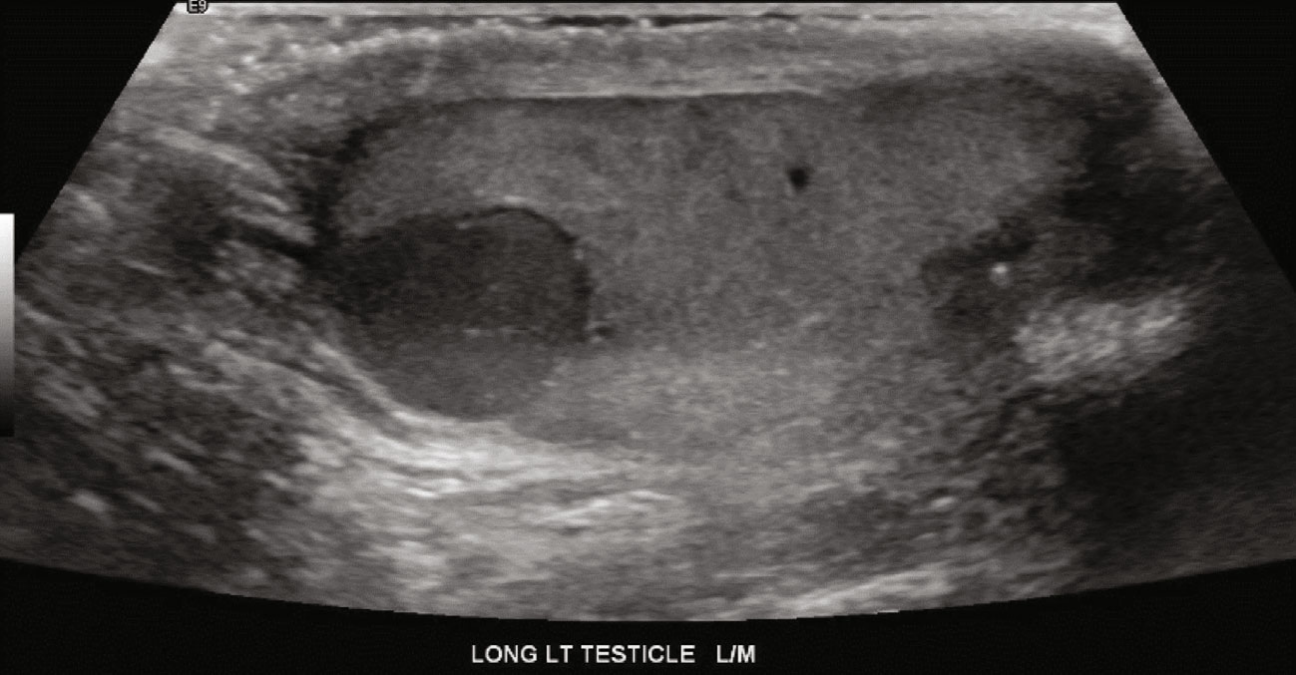
Scrotal ultrasound demonstrating left testis mass.

A 27-year-old male presented with intermittent left testicular pain and abnormal imaging following a motorcycle accident in which he sustained left leg injuries necessitating above-knee amputation. Computerized tomography (CT) and magnetic resonance imaging (MRI) from that hospitalization noted a left testis abnormality. A scrotal ultrasound was performed which demonstrated a 1.3 × 0.8 × 1.2 cm lobular hypoechoic mass in the left testicle with vascular flow suspicious for malignancy (Figure 1). He had no history of cryptorchidism, inguinal hernias, or congenital limb or orofacial abnormalities.

On examination, he had bilaterally descended and mildly atrophic testes with a firm palpable mass in the left testis. No hernias were appreciated. Preoperative serum tumor markers were all within normal limits: alpha fetoprotein 1.1 ng/mL, beta-human chorionic gonadotropin < 1 mIU/mL, and lactate dehydrogenase 128 U/L.

Repeat CT scan did not demonstrate any evidence of metastatic disease or retroperitoneal lymphadenopathy but revisualized the left testis mass. Due to concerns for malignancy, he underwent a left inguinal orchiectomy, with an uneventful postoperative course.

**Figure 2:**
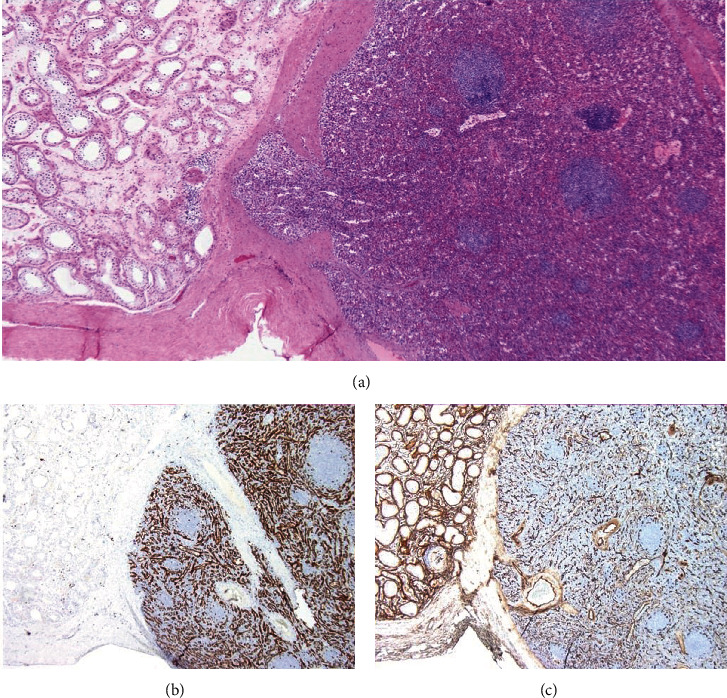
Pathologic specimen demonstrating (a) benign splenic tissue separated by fibrous cap, (b) CD8 staining of littoral cells, and (c) CD34 staining of endothelial cells.

Pathology of the “beefy” red mass measuring 1.4 × 1.2 × 1.1 cm was consistent with SGF, with a fibrous capsule separating benign splenic tissue from testicular and epididymal parenchyma. Histologic examination revealed a well-circumscribed mass with a thick fibrous capsule, composed of capillary vessels, sinusoids, and large, thick-walled vessels with interspersed lymphoid follicles (Figure 2). Background testis showed no pathologic abnormality. CD34 was used to highlight the endothelial cells and CD8 for littoral cells.

## 3. Discussion

Less than 200 cases of SGF have been identified since the entity was first described in the 1880s [1, 2]. Of the reported cases, 95% occurred in men (16 : 1 M:F ratio), though the gender discrepancy may be related to intraabdominal ovaries being less easily examined. The majority of cases are diagnosed in patients under age 30 and are predominantly left-sided [1, 2].

SGF is classified into two subtypes: continuous and discontinuous. Continuous SGF describes a band of tissue made of splenic parenchyma, fibrotic material, or both that extends from the spleen to the scrotum. Discontinuous SGF refers to splenic rests in a gonad that do not have any anatomic connection back to the spleen. Continuous SGF accounts for 55% of cases and is associated with cryptorchidism and a fivefold increase in concomitant limb or orofacial malformations [3, 4]. Discontinuous SGF is typically not associated with other congenital abnormalities.

The mechanism of SGF is not well-understood and it has even been proposed that the two subtypes may arise through entirely different processes. Le Roux and Heddle suggested that discontinuous SGF may be generated like accessory spleens, which form when the splenic anlage fails to fuse around week 5 of development [5]. During weeks 5-8 of embryologic development, the splenic anlage is in close proximity to the left urogenital fold. Primitive gonadal structures will then start their descent around week 8 of life. Limb buds and Meckel's cartilage (mandible precursor) are both also developing during weeks 4-8. It has been proposed that a teratogenic insult during this interval may result in SGF combined with peromelia and orofacial anomalies [6].

SGF is often discovered as an incidental testicular mass during orchidopexy or inguinal hernia repair. It can also present as scrotal swelling. Previous reports have described worsening scrotal swelling with malaria, mononucleosis, mumps, and hematologic malignancies that can affect splenic tissue [3].

There have been four case reports of men diagnosed with SGF and concomitant testis cancer after orchiectomy. However, all four of these men had a history of cryptorchidism which independently confers an increased risk of testis cancer. There is no established link between SGF alone and testis cancer [3].

SGF can present similarly to testis cancer and diagnosing SGF prior to orchiectomy becomes immensely challenging, particularly in discontinuous cases. Imaging is largely inconclusive and there is overlap between SGF imaging findings and those of testicular malignancies. On ultrasound, SGF may present as an encapsulated homogenous and hypoechoic lesion with internal vascularity on Doppler, which is consistent with our patient's findings [7, 8]. This is nonspecific, however, as testis cancers can present identically. On noncontrast CT images, normal splenic tissues (including accessory spleens) have attenuation values in the 40-60 Hounsfield unit (HU) range and appear homogenous [8]. Normal splenic white pulp and red pulp have different vascular architecture, and contrast is initially transmitted at different speeds in each circulatory system. On CT with contrast, the differential flow between the white vs. red pulp circulation leads to heterogenous enhancement in the arterial/early venous phase. The contrast transmission ultimately evens out and white and red pulp enhance homogenously during late venous/delayed phase CT images. Again though, testicular malignancies can have variable vascularity.

**Figure 3:**
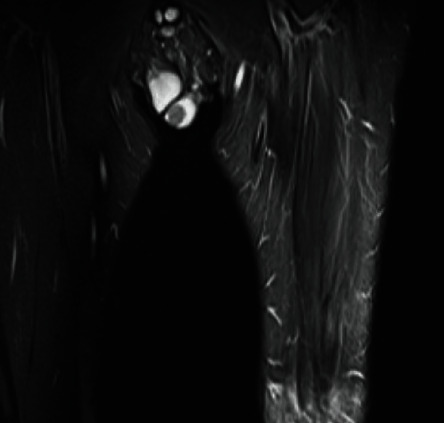
MRI with T2 imaging demonstrating hypointense nodule on the left testis.

MRI contrast patterns for splenic tissue mirror CT patterns in terms of heterogenous enhancement during arterial phases and homogenous enhancement during venous/delayed phase. On T1 imaging, splenic tissue tends to have a lower signal intensity than liver/muscle; T2 splenic tissue has a higher intensity than liver/muscle parenchyma [8]. Our patient's testis abnormality appeared as a T2 hypointense lesion on MRI of his femur that was performed to assess lower extremity sequelae postamputation (Figure 3). His testis mass appears to have a similar intensity on T2 images compared to muscle, though per radiology his lower extremity muscles on this scan were edematous.

Radiocolloid spleen scintigraphy (99mTc-sulphur colloid spleen-liver scan) has also been utilized in two case reports to identify ectopic splenic tissue suspicious for SGF and avoid surgical intervention. One case report involved a 20-year-old female with orofacial anomalies and a 6 cm intraabdominal mass with biopsy pathology consistent with lymphoid cells [9]. The second case involved an infant with hemimelia and undescended left testicle [6]. The nuclear scan was able to demonstrate a continuous tail from his inferior spleen down to the undescended testicle. In cases of discontinuous SGF, where diagnosis may be more challenging, nuclear imaging may suggest the diagnosis, although this is unlikely to avoid surgical removal to definitively exclude underlying malignancy.

## 4. Conclusions

SGF is a rare entity and remains difficult to identify preoperatively as it shares clinical and imaging findings with testicular malignancies. Given case reports of concomitant testis cancer, we favor proceeding with orchiectomy when a suspicious testicular mass is noted on imaging.
